# Night Sweats as a Prominent Symptom of a Patient Presenting with Pulmonary Embolism

**DOI:** 10.1155/2015/841272

**Published:** 2015-05-11

**Authors:** Attila Feher, Saif A. Muhsin, Anna M. Maw

**Affiliations:** Department of Medicine, Weill Cornell Medical College, New York, NY 10065, USA

## Abstract

Pulmonary embolism is a common, potentially fatal disease. Making the correct diagnosis early can significantly reduce mortality and morbidity. We report the first case of drenching night sweats as one of the presenting symptoms of submassive pulmonary embolism. One week after undergoing laparotomic sigmoidectomy for diverticulitis, our patient started to experience drenching night sweats and pleuritic back pain. CT identified bilateral main stem pulmonary artery emboli, and treatment was initiated with subcutaneous enoxaparin injections. Imaging and laboratory workup failed to reveal any other explanation for the night sweats. Patient was discharged on rivaroxaban, and he reported complete resolution of symptoms upon the 3-month follow-up visit and 9-month follow-up call. Based on our case we propose night sweats to be a potential presenting symptom of pulmonary embolism. Our observation can help make an earlier diagnosis of pulmonary embolism.

## 1. Introduction

Massive pulmonary embolism (PE) is a life threatening condition; early diagnosis and early anticoagulation can reduce the risk of adverse clinical outcomes [1, 2]. The most common presenting symptoms of PE are resting or exertional dyspnea, pleuritic chest pain, cough, lower extremity pain/swelling, and wheezing [3]. Here we report a case of PE that presented with back pain and profuse night sweats.

## 2. Case Presentation

A 60-year-old Caucasian man presented with 2 weeks of back pain with drenching night sweats 20 days after undergoing laparotomic sigmoidectomy for severe diverticulitis. One week after his discharge, the patient developed right sided lower back pain and severe drenching night sweats that prompted him to change his clothing and bed sheets multiple times every night. The patient described the pain as a 7/10 throbbing, sharp pleuritic pain that was better in supine position and was initially responsive to nonsteroid anti-inflammatory drugs. The patient experienced mild shortness of breath on exertion. He denied any nausea, vomiting, chest pain, or chills or fevers. He had a 12-pound weight loss starting 3 weeks prior to presentation. His medications were silodosin, atorvastatin, diazepam, docusate, and as needed acetaminophen, naproxen, and oxycodone. Patient never smoked and lived an active life prior to his surgery.

On physical examination he was afebrile, resting in bed comfortably. Heart rate was 100 beats/minute, blood pressure was 144/91 mmHg, respiratory rate was 20 breaths/minute, and oxygen saturation level was 97% on room air. He had no palpable lymph nodes and had bibasilar dullness to percussion with decreased breath sounds. Auscultation of the heart revealed tachycardia, but regular and normal heart sounds. The right lower back was tender to palpation. The abdominal surgical scar was intact with no signs of inflammation.

**Figure 1:**
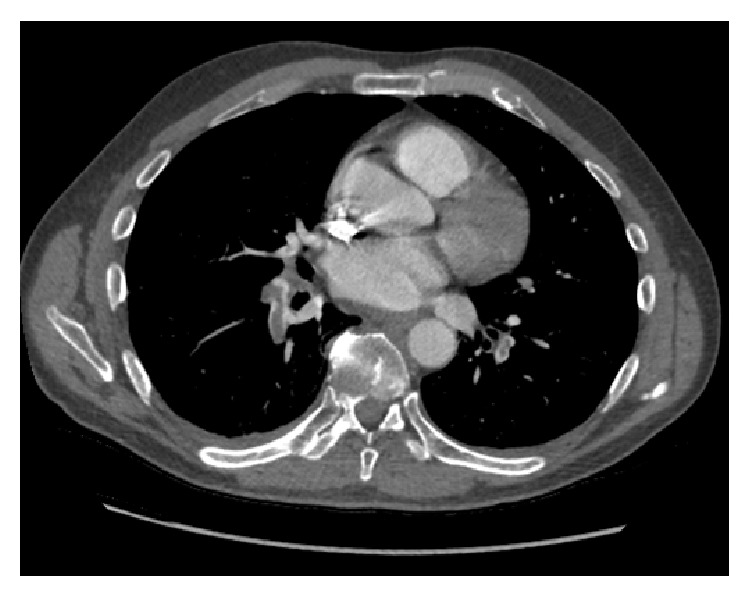
Chest CT showing bilateral filling defects involving the right and left main pulmonary arteries with extension to the segmental branches of the right middle and bilateral lower lobes.

The laboratory findings were hemoglobin level: 13.1 mmol/L; white blood cell count: 10.4 × 10^3^/mL; platelet count: 354 × 10^3^/mL; troponin: 0.01 ng/mL. Basic metabolic profile values were all within normal limits. Our initial differential diagnosis list included PE, pneumonia, pericarditis, and pneumothorax. As the patient presented with pleuritic back pain after recent surgical intervention, PE was very high on our differential diagnosis. Chest radiography revealed bilateral basilar opacities and no pneumothorax. CT imaging of the chest demonstrated PE involving the main pulmonary arteries (Figure 1). Transthoracic echocardiogram showed no sign of pericardial effusion.

Therapy was initiated with subcutaneous enoxaparin injections. The back pain resolved after the first day, but night sweats continued. Further diagnostic workup showed elevated erythrocyte sedimentation rate (ESR): 53 mm/h, C-reactive protein (CRP): 19.34 mg/dL, normal thyroid-stimulating hormone, and normal procalcitonin. CT of abdomen and pelvis was performed to rule out abdominal abscess and showed postsigmoidectomy state with intact anastomosis. MRI of the spine revealed no epidural mass or collection. Based on clinical guidelines the patient was discharged home on 3 months of rivaroxaban treatment [4]. Patient's night sweats completely resolved after discharge, verified by a 3-month follow-up visit and by a 9-month follow-up call.

## 3. Discussion

Fever and systemic inflammation have been long recognized as components of acute PE syndromes [5, 6]. Patients with venous thromboembolism demonstrate increased levels of proinflammatory cytokines including interleukin-6, interleukin-8, and TNF-alpha [6–8] and the formation of blood clots has been shown to lead to thrombus fragmentation and generation of proinflammatory fibrin breakdown products [9]. The release of cytokines causes a temporal increase in the body's inner temperature threshold, the thermoneutral zone, resulting in subsequent rise in the body's temperature. Night sweats occur when the levels of these mediators and the thermoneutral zone return to normal, and the body makes an attempt to cool down the core temperature [10]. Another potential mechanism for PE associated night sweats could be direct stimulation of sweat glands by cytokines as eccrine sweat glands have been reported to express receptors for most PE associated cytokines [11–13].

Although increased inflammatory state is often published as a prominent feature of PE, to our knowledge, night sweats have not been previously reported as a key feature of PE. Occasional case reports have mentioned night sweats as part of PE syndromes, but these attributed night sweats to the presence of infection [14, 15] or an underlying malignancy [16]. In a recent review article, Mold et al. reported a comprehensive list of clinical conditions that have been associated with night sweats and PE is not listed among them [10].

After initiation of anticoagulation therapy with enoxaparin and continuation with rivaroxaban, our patient's night sweating episodes disappeared; this suggests that this symptom was related to PE. The precise mechanism by which PE would lead to night sweats is currently unclear, but we can postulate that the release of proinflammatory cytokines can lead to generalized inflammation, detected by ESR and CRP in our patient.

Based on the presented case, night sweats can be among the presenting symptoms of PE; our observation can help make an earlier diagnosis of pulmonary embolism. Prompt diagnosis of PE is critical as timely initiation of therapy can be lifesaving in many cases.
